# Sexual dimorphism and acute stress modulation of infralimbic-posterior hypothalamic synaptic transmission

**DOI:** 10.3389/fncel.2026.1659293

**Published:** 2026-04-10

**Authors:** Yesenia Rivera-Escobales, Danisha N. Hernández-Crispín, Jaileene Pérez-Morales, María Colón, James T. Porter

**Affiliations:** 1 Department of Basic Sciences, Ponce Research Institute, Ponce Health Sciences University, Ponce, Puerto Rico; 2 Division of Oncological Sciences, Knight Cancer Institute, Oregon Health & Science University, Portland, OR, United States

**Keywords:** infralimbic cortex, posterior hypothalamus, restraint stress, sex differences, stress adaptation, synaptic plasticity

## Abstract

Acute stress engages neural circuits that coordinate autonomic and neuroendocrine responses, including projections from the infralimbic cortex (IL) to the posterior hypothalamic nucleus (PH). Although both regions are activated during stress, the synaptic mechanisms underlying IL-to-PH communication remain poorly understood. Here, we combined optogenetics with whole-cell patch-clamp electrophysiology to determine how acute restraint stress alters excitatory synaptic transmission from IL to PH neurons in adult male and female rats. IL afferents formed functional glutamatergic synapses onto PH neurons in both sexes, characterized by short-term facilitation and a high AMPA/NMDA ratio. However, females exhibited smaller optically evoked excitatory postsynaptic currents (EPSCs) in response to single or burst stimulation across multiple holding potentials. NMDA receptor-mediated EPSCs and NMDAR-predominant spontaneous EPSCs also displayed sex differences, with females showing smaller and faster synaptic currents. When data were collapsed across sex, acute restraint stress enhanced NMDAR-mediated synaptic currents at IL-to-PH synapses while reducing the amplitude of NMDAR-predominant spontaneous EPSCs without altering their frequency. Together, these findings reveal sex-dependent differences in excitatory IL-to-PH synaptic signaling and suggest that acute stress preferentially modulates NMDAR-mediated transmission in this pathway. These results highlight dynamic postsynaptic mechanisms that shape prefrontal-hypothalamic communication during acute stress.

## 1 Introduction

Stress exposure triggers a cascade of autonomic and neuroendocrine responses via brain regions like the posterior hypothalamic nucleus (PH). Adaptation to stress responses is important for proper physiological and psychological function. Experimental evidence suggests that the PH function is important for stress adaptation. Animal studies show increased neuronal activity in the PH of male rats in response to chronic stress (Flak et al., 2012). Studies show that PH activity is necessary to adapt the hypothalamic–pituitary–adrenal (HPA) axis response to stress (Myers et al., 2016; Nyhuis et al., 2016). Blocking GABA receptors in the PH before exposure to restraint stress enhanced the adrenocorticotropic-releasing hormone response to stress, increasing corticosterone plasma levels during restraint stress (Myers et al., 2016). Pharmacological inhibition of the PH has been shown to decrease adrenocorticotropin-releasing hormone and corticosterone plasma levels (Myers et al., 2016; Nyhuis et al., 2016). More importantly, inhibition of the PH before stress exposure disrupts HPA axis stress adaptation (Nyhuis et al., 2016).

Another important brain region in charge of the stress response is the infralimbic cortex (IL). Optogenetic stimulation of IL during restraint stress reduces corticosterone plasma levels in male rats. However, it does not affect corticosterone levels of female rats (Wallace et al., 2021). The IL innervates several brain regions involved in the autonomic response, including the PH (Myers et al., 2016; Vertes, 2004; Wood et al., 2019) Reports supporting an association between IL and PH show that innervation from IL to PH is mostly glutamatergic (Myers et al., 2016) and that IL stimulation increases PH neuronal activity (Wood et al., 2019). Likewise, both brain regions are simultaneously activated in response to stress (Flak et al., 2012). Recent studies show that optogenetic stimulation of IL terminals in the PH during restraint stress does not influence corticosterone plasma levels in male rats but increases the corticosterone response in females post-stress (Schaeuble et al., 2024).

Despite the evidence supporting a role for the PH in regulating the stress response, whether synaptic plasticity in the PH contributes to stress adaptation remains to be investigated. In addition, how acute stress affects synaptic transmission between IL and PH has not been explored. In the present study, we aimed to examine how restraint stress impacts synaptic transmission in the PH of adult male and female rats. Using optogenetic recording in PH brain slices, we found that baseline sex differences in NMDA signaling shape how IL inputs are integrated in the PH. Moreover, acute restraint stress selectively enhances NMDAR-mediated transmission at IL-to-PH synapses.

## 2 Materials and methods

### 2.1 Subjects

All animal procedures were approved by the Institutional Animal Care and Use Committee of the Ponce Health Sciences University in compliance with the National Institute of Health guidelines for the care and use of laboratory animals. In-house-bred adult male (280–310 g) and female (220–286 g) Sprague Dawley rats were used for all experiments. Animals were housed in groups of three males or three females in a 12/12 h light/dark cycle with free access to food and water.

### 2.2 Stereotaxic injections

Rats were anesthetized with isoflurane (5% for induction, 1–2% for maintenance; flow rate: 0.5 L/min) and secured in a stereotaxic apparatus. Ophthalmic ointment was applied to their eyes, and the scalp fur was trimmed. The scalp was disinfected with Betadine. Unilateral craniotomies were drilled above the target regions. The virus was infused using a programmable syringe pump with a Hamilton syringe. The IL was infused unilaterally with 0.5 μL of AAV-CaMKIIa-hChR2(H134R)-EYFP virus at a rate of 0.05 μL/min, using the following coordinates (relative to bregma): anteroposterior (AP), 2.5; mediolateral (ML), 0.5; dorsoventral (DV), −5.0 for females rats and anteroposterior (AP), 2.5; mediolateral (ML), 0.6; dorsoventral (DV), −5.7 for males rats. Injectors were left at the target coordinates for 10 min after infusion to allow for diffusion. The syringe was then removed, and the incision was closed with sutures. Once closed, VetBond and antibiotics were applied to the scalp. Ketorolac (5 mg/kg) was administered immediately after surgery. Animals were left in their home cages for 8–12 weeks to allow expression of the virus.

### 2.3 Ex vivo electrophysiology

Adult male and female rats (15–22 weeks old) were exposed to restraint stress for 30 min using a disposable rodent restrainer (DecapiCone®; Cat. No. DC-200). Control groups of non-stressed females and males were kept in their home cages until sacrifice. Immediately after the restraint stress, rats were deeply anesthetized with pentobarbital (65 mg/kg), perfused transcardially with ice-cold N-methyl-D-glucamine (NMDG) based artificial CSF (ACSF), and decapitated. Brains were quickly removed to collect 300 μm coronal slices of the PH using a Vibratome 1000 Plus (Vibratome). We used a modified NMDG-based ACSF to obtain healthy brain slices from adult animals. The composition of the NMDG-based ACSF was the following: 93 mM NMDG, 2.5 mM KCl, 1.2 mM NaH2PO4, 30 mM NaHCO3, 20 mM HEPES, 25 mM glucose, 5 mM sodium ascorbate, 2 mM thiourea, 3 mM sodium pyruvate, 10 mM MgSO4, and 0.5 mM CaCl2. The PH slices were initially incubated at 33 °C in NMDG ACSF for 10 min before being transferred to an additional 1-h incubation in modified HEPES ACSF at room temperature (21–23 °C). The composition of the modified HEPES ACSF was the following: 92 mM NaCl, 2.5 mM KCl, 1.2 mM NaH2PO4, 30 mM NaHCO3, 20 mM HEPES, 25 mM glucose, 5 mM sodium ascorbate, 2 mM thiourea, 3 mM sodium pyruvate, 2 mM MgSO4, and 2 mM CaCl2. Then, PH slices were transferred, submerged in the recording chamber, and perfused at 2–3 mL/min with room temperature ACSF. The composition of the recording ACSF was the following: 126 mM NaCl, 3 mM KCl, 1.25 mM NaH2PO4, 1 mM MgSO4, 26 mM NaHCO3, 20 mM glucose, and 2 mM CaCl2, and bubbled with 95% O2 and 5% CO2. The neurons were visualized with infrared video microscopy using a 60X water-immersion objective on an upright Eclipse FN1 Nikon microscope. Whole-cell recordings were done with glass pipettes with a resistance of 2.5–5 MΩ and filled with cesium gluconate internal solution containing the following: 10 mM CsCl, 130 mM CsOH, 10 mM HEPES, 130 mM gluconic acid, 5 mM biocytin, 4 mM adenosine triphosphate, 0.3 mM guanosine triphosphate, and 11 mM cesium-ethylene glycol-bis(2-aminoethylether)-N,N,N′, N′-tetraacetic acid (Cs-EGTA), 5 mM QX-314; pH was adjusted to 7.3 with CsOH (300 mOsm). After establishing a whole-cell voltage-clamp recording, membrane resistance, membrane capacitance, and access resistance were measured. We monitored the access resistance and excluded recordings in which the access resistance was higher than 25 MΩ from analysis. Recordings were filtered at 4 kHz, digitized at 10 kHz, and saved to a computer using pCLAMP v11.2 (Molecular Devices, San Jose, CA). Recordings were analyzed using Clampfit v11.2 (Molecular Devices, San Jose, CA). Raw data were low-pass filtered using an 8-pole Bessel filter with a −3 dB cutoff frequency of 1,400 Hz, applied across the full trace. Baseline correction was conducted by subtracting the mean of a region before optical stimulation. For spontaneous excitatory post-synaptic currents (sEPSCs), the baseline was corrected by subtracting the mean of the entire trace or a manually selected region in traces with artifacts. To assess recording quality across groups, baseline noise was quantified from sEPSC recordings. For each cell, three short event-free baseline windows were selected throughout the recording. Noise was quantified as the standard deviation of the holding current within each window. Standard deviation values were averaged per cell to generate a single baseline noise value per neuron, which was used for group-level statistical comparisons across sex and stress conditions. We did not detect differences in the standard deviation of the noise between groups (Supplementary Figure 1). Event detection of the sEPSCs was performed using the threshold trace function with a negative-going polarity. Trigger and re-arm parameters were set, with pre-trigger and post-trigger durations of 10 ms and 60 ms, respectively. Events shorter than two ms were excluded using the event rejection setting.

### 2.4 AMPA receptor (AMPAR) and NMDA receptor (NMDAR) currents

**Figure 1:**
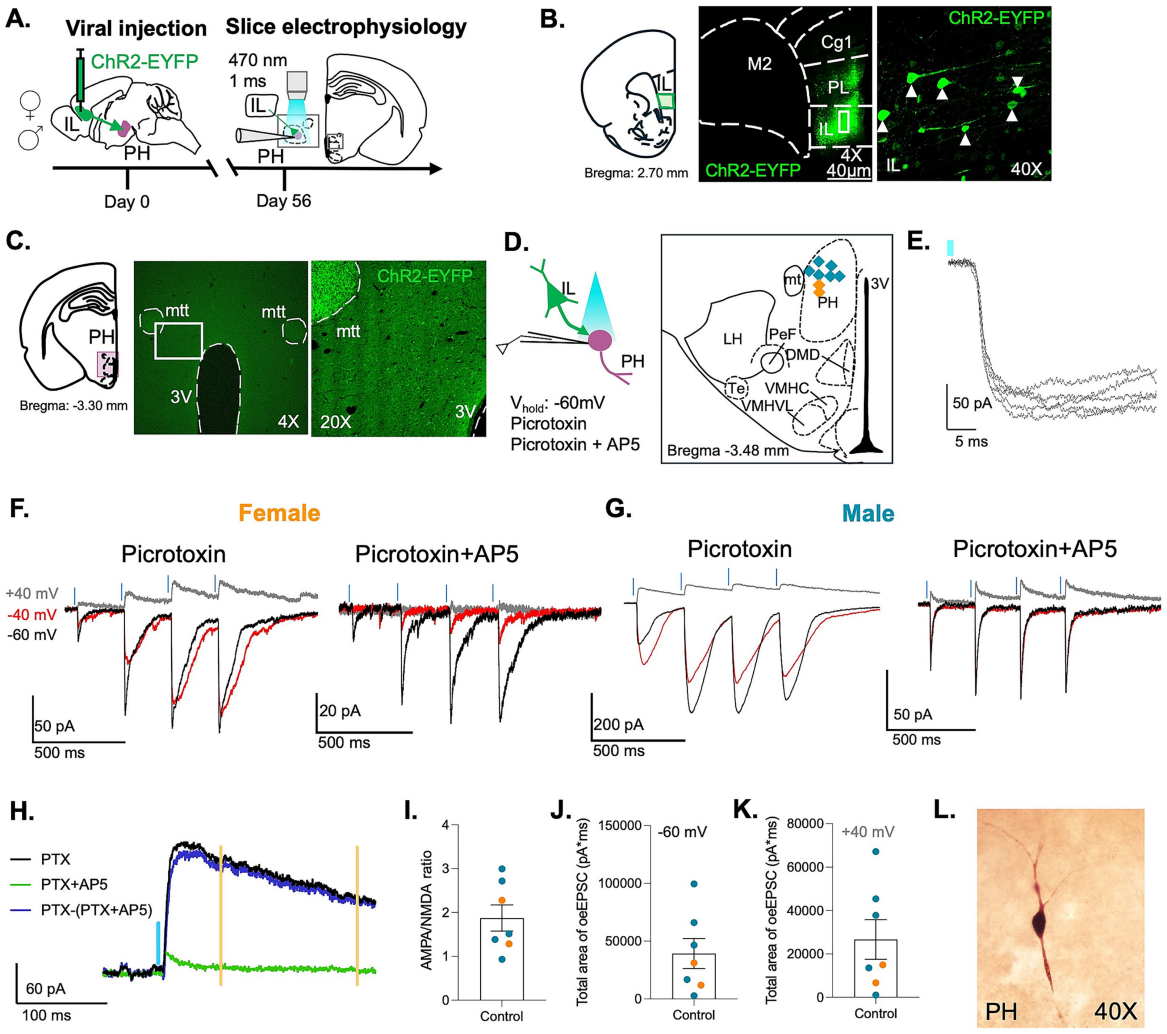
IL synaptic input onto PH neurons. **(A)** Timeline of experiments. Male and female rats received stereotaxic injections of an AAV-CaMKIIa-hChR2(H134R)-EYFP virus. Whole-cell patch clamp recordings of PH neurons with IL inputs were performed at least 8 weeks after injection. **(B)** Representative images of ChR2 expression in pyramidal neurons in the IL. **(C)** ChR2 expression in PH. **(D)** Schematic depicting optogenetic stimulation of IL terminals with blue light (470 nm, 1 ms) and recording of optically evoked EPSCs in PH neurons. Illustration of the recording site in the PH with locations of recorded neurons illustrated as orange (females) and blue (males) circles. **(E)** Overlapping optically evoked EPSCs in a PH neuron showing a lack of jitter in EPSC onset. **(F, G)** Representative traces of optically evoked EPSCs in female and male rats with picrotoxin alone and after adding AP5 at holding potentials of −60 (black), −40 (red), and +40 mV (gray). **(H)** Traces showing a lack of AMPAR currents when the NMDAR current was measured between the yellow lines, 70–230 ms after optical stimulation, indicated by the blue line. **(I)** AMPA/NMDA ratio of optically evoked IL inputs. **(J)** Total area of optically evoked EPSC recorded at −60 mV. **(K)** Total area of optically evoked EPSC recorded at +40 mV. 3 rats/7 cells; female (orange), male (blue). **(L)** Image of a neuron recorded in PH.

AMPAR and NMDAR-mediated EPSCs in PH neurons were measured in response to the optical stimulation of ChR2-expressing IL axons with a 470-nm light-emitting diode (LED; X-CITE Xylis XT720S-0159, Excelitas Technologies) through the 60X objective centered at the soma of the patched neuron with light intensity of 15%. Picrotoxin (10 μM) was added to the bath to block GABA_A_-mediated currents. To evoke synaptic responses in the PH via photo-stimulation of IL fibers, we illuminated slices at 20 s intervals with trains of four 1-ms light pulses separated by 250 ms (4 Hz). For the initial characterization of IL-to-PH synaptic transmission (Figure 1), synaptic responses were sequentially recorded from the same PH neurons at holding potentials of −60 mV, −40 mV, and +40 mV in the presence of picrotoxin. Slices were then perfused with the NMDAR antagonist DL-AP5 (100 μM) while maintaining picrotoxin in the bath, and synaptic currents were recorded again at the same holding potentials to isolate AMPAR-mediated responses. In all subsequent experiments, recordings were performed in the presence of picrotoxin alone; therefore, EPSCs represent mixed AMPAR/NMDAR synaptic responses. The recordings of the sEPSCs include neurons with IL input and neurons without IL input. A total of 163 neurons were recorded from the PH.

### 2.5 Immunohistochemistry and imaging

Brains were fixed with 10% Formalin and sliced with a vibratome. Injections of AAV-CaMKIIa-hChR2(H134R)-EYFP in the IL were confirmed using a Nikon Confocal Microscope A1 (Ver. 4.10). Identification of biocytin-filled recorded neurons was done using the avidin-biotin-complex method (ABC; Vectastain ABC kit PK-6100). Recorded 300 μm PH slices were fixed in 10% Formalin at 20 °C and placed afterward in PBS 10 mM. On the first day of staining, slices were washed three times using PBS 10 mM containing 0.25% Triton X-100, then incubated for 30 min in 3% H_2_O_2_. Slices were washed with PBS 10 mM for two rounds of 10 min before adding the ABC solution and being left to shake for 2 h before an overnight incubation at 4 °C. On the second day, tissues were washed twice with PBS 10 mM for 20 min, followed by an hour of washing. Then, tissues were incubated in DAB solution for 15 min. Slices were then exposed to DAB +0.3% H_2_O_2,_ and when axons were sufficiently dark, the reaction was stopped with 10 mM PBS. The tissues were washed with PBS 10 mM for two rounds of 10 min and mounted on slides using Cytoseal XYL (Richard-Allan Scientific 8312-4) and a coverslip. Images were taken using a Nikon Eclipse E200 Microscope.

### 2.6 Statistical analysis

Statistical analyses were performed using R (R Foundation for Statistical Computing) with RStudio (version 2025.09.2+418). Data were analyzed to assess the effects of sex (female, male), stress (no stress, stress), and their interaction on the dependent variable. Normality of residuals was assessed using the Shapiro–Wilk test, performed separately for each Sex × Stress group. Because residuals deviated from normality in one or more groups, a non-parametric factorial approach was employed. A two-way ANOVA with main effects of sex and stress, as well as their interaction, was assessed using the Aligned Rank Transform (ART) ANOVA (Wobbrock et al., 2011) implemented with the ARTool package. Estimated marginal means (EMMs) were computed and visualized for each factor and the interaction of sex and stress. When a significant sex × stress interaction was detected, post-hoc analyses were conducted on the ART-transformed data. Pairwise comparisons between all sex × stress groups were performed using Tukey’s adjustment to control for multiple comparisons. Post-hoc results are reported as estimated differences between group means, associated standard errors, degrees of freedom, t-statistics, and adjusted *p*-values. Descriptive statistics are reported as mean ± standard deviation, calculated from the raw (untransformed) data for each sex × stress group. Raw data are visualized using boxplots, illustrating the median (maximum and minimum) and individual data points for each group. Statistical significance was set at *p* ≤ 0.05. All analyses were performed in R using ARTool, emmeans, ggplot2, flextable, and officer packages. Detailed statistical results corresponding to each figure are reported in the Supplemental Statistical Table. Data in figures are shown as boxplots with median and interquartile range. Individual observations are plotted as points.

## 3 Results

### 3.1 IL synaptic input onto PH neurons

Although studies show that the IL innervates the PH (Myers et al., 2016; Vertes, 2004; Wood et al., 2019), previous studies have not examined the properties of IL synapses onto PH neurons. To confirm and characterize IL excitatory connections with PH neurons, we injected a channelrhodopsin-expressing viral vector into the IL of adult male and female rats. After 8–12 weeks to allow for channelrhodopsin expression and anterograde transport to synaptic terminals in the PH (Figures 1A–C), we prepared acute brain slices containing the PH. We performed whole-cell patch-clamp recordings while optically stimulating IL terminals. Although the infected neurons were centered in IL, there was some labeling in the prelimbic cortex (Figure 1B). Previous studies indicate that the IL extensively innervates the PH, whereas the prelimbic cortex sends limited projections to the PH (Hurley et al., 1991; Vertes, 2004). Therefore, most of the synaptic inputs evaluated in this study likely originate in the IL. GABAergic transmission was blocked using picrotoxin (10 μM) to isolate EPSCs evoked by optical stimulation of IL terminals (Figure 1D). The latency of optically evoked EPSCs was short and consistent, with a low jitter, consistent with monosynaptic input (Figure 1E). Brief (1 ms) blue light pulses reliably evoked inward EPSCs at a holding potential of −60 mV in PH neurons from both sexes (Figures 1F,G), confirming functional connectivity between IL and PH neurons. To investigate the short-term plasticity of presynaptic release properties, we delivered a train of four 1 ms light pulses at 15% light intensity and analyzed the amplitude of EPSCs across the train at a holding of −60 mV (Figures 1F,G). In both sexes, we observed short-term facilitation of the second EPSC. These results suggest that the glutamate release probability at IL-to-PH synapses is initially relatively low but shows stronger responses to bursts of synaptic inputs.

To examine the relative postsynaptic receptor composition, we isolated AMPA receptor and NMDA receptor-mediated components of the EPSC by recording at −60, −40, and +40 mV in the absence and presence of the NMDA receptor antagonist DL-AP5 (100 μM). AMPA and NMDA receptors contributed to the currents at all three holding potentials. We calculated the AMPA/NMDA ratio (−60 mV/+40 mV) for each cell. The AMPA component was measured as the peak of EPSC recorded at −60 mV in the presence of picrotoxin and AP5. In contrast, the NMDA receptor component was measured from the EPSC recorded at +40 mV during a delayed window (70–230 ms after stimulation) when the AMPA receptor-mediated currents had decayed (Figure 1H). Neurons in the PH of male and female rats showed a high AMPA/NMDA ratio (Figure 1I). We also quantified the total synaptic current produced by the burst of four stimuli (Figures 1J,K) in recorded PH neurons (Figure 1L).

For the initial characterization of IL synaptic input to PH neurons, the AMPAR component of the EPSC was isolated by blocking NMDARs with the antagonist AP5. In subsequent experiments, AP5 was omitted to allow recordings from multiple neurons within the same slice, as incomplete washout of AP5 can affect successive recordings. Under these conditions, optically evoked EPSCs therefore reflect mixed AMPAR/NMDAR synaptic responses, with the relative contribution of each receptor type depending on the holding potential.

### 3.2 Females receive smaller IL-to-PH synaptic inputs at −60 mV

**Figure 2:**
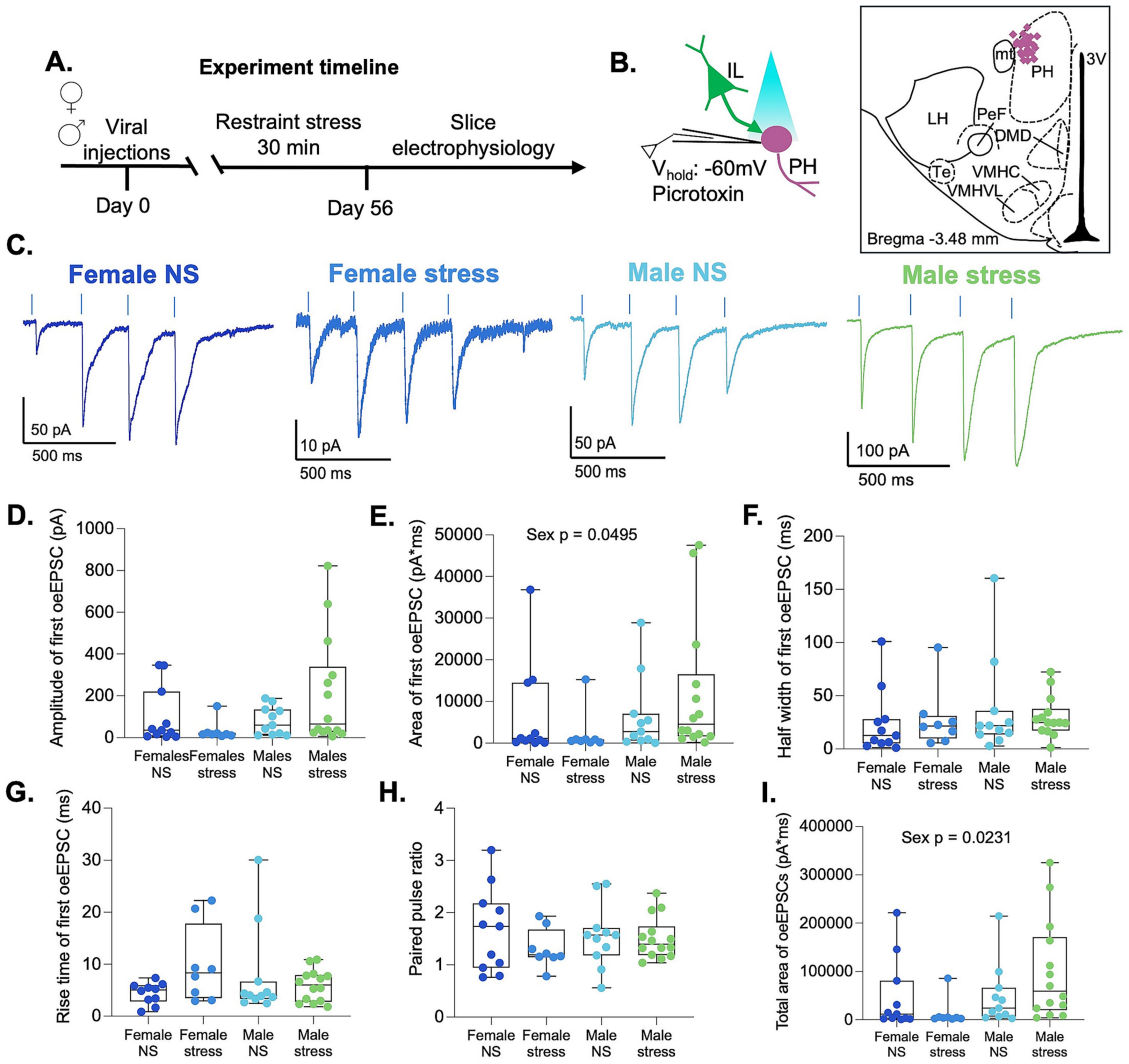
IL-to-PH synaptic strength is weaker in females. **(A)** Timeline of experiments. **(B)** Schematic depicting optogenetic stimulation of IL terminals and recording of PH neurons. Illustration indicating recorded neurons as purple diamonds in the PH. **(C)** Representative traces of optically evoked EPSCs in female and male rats. **(D)** Amplitude **(E)**, area **(F),** half-width **(G)**, and rise time of optically evoked EPSCs recorded at −60 mV in response to the first stimulus. **(H)** PPR of optically evoked EPSCs. **(I)** Analysis of the total area of optically evoked EPSC across a train of four stimuli in females and male rats. Female no-stress (NS) group *n*: 4 rats/11 cells. Female stress group *n*: 3 rats/8 cells. Male no-stress (NS) group *n*: 4 rats/11 cells. Male stress group *n*: 4 rats/14 cells.

Experimental evidence suggests that PH activity is necessary to adapt the HPA axis response to stress (Myers et al., 2016; Nyhuis et al., 2016). However, whether acute stress induces synaptic plasticity in the PH that could contribute to stress adaptation remains to be elucidated. To address this, we exposed male and female rats to restraint stress for 30 min to examine whether acute stress induces synaptic plasticity within the IL-to-PH synapses (Figure 2A). Using whole-cell patch-clamp electrophysiology in acute PH brain slices, we measured optically evoked mixed AMPAR/NMDAR EPSCs at −60 mV (light duration 1 ms; Figures 2B,C). Since the data did not pass normality tests, we used an ART ANOVA to compare the optically evoked EPSCs with sex and stress as factors. Analysis of the first EPSC showed that the amplitude was similar in all groups. However, the total charge transfer (area) showed a main effect of sex [*F* (1,40) = 4.10, *p* = 0.049] with no effect of stress, indicating that the strength of IL synaptic input was smaller in females and was not altered by acute stress (Figures 2D,E). The analysis revealed no differences in EPSC half-width or rise time, suggesting that acute stress did not alter the kinetics of the EPSCs in either sex (Figures 2F,G). In addition, the paired-pulse ratio (PPR = amplitude of second EPSC/amplitude of first EPSC) was similar in all groups, suggesting that presynaptic release properties were similar in both sexes and were not altered by acute stress (Figure 2H). In all groups, the ratio was greater than one, indicative of paired-pulse facilitation. To examine the effect of a burst of inputs, we delivered a train of four 1 ms light pulses at 15% light intensity and analyzed the total charge transfer of the four EPSCs across the train (Figure 2I). We found a main effect of sex [*F* (1,40) = 5.58, *p* = 0.023] with smaller total current in the females. These results indicate that single or bursts of IL inputs produce larger synaptic responses in males. Overall, acute stress did not alter the strength of synaptic transmission in IL-to-PH synapses in either sex at this holding potential.

Some averaged EPSC traces displayed an apparent second peak, suggesting the possibility of feedforward excitation or recruitment of multiple IL inputs with distinct synaptic kinetics. However, overlaying individual traces revealed a highly consistent EPSC onset with minimal latency jitter, arguing against polysynaptic activity (Supplementary Figure 2). This observation is consistent with previous studies demonstrating sparse local connectivity within hypothalamic nuclei (Averbeck and Murray, 2020; Burdakov and Karnani, 2020; Razenberg et al., 2024; Reppucci and Petrovich, 2016; Shao et al., 2022). Instead, the apparent second peak likely reflects a dual decay phase present in a subset of cells, possibly resulting from activation of IL inputs that generate EPSCs with different decay kinetics. These differences in decay kinetics may reflect varying contributions of NMDAR-mediated currents at different synapses. To determine whether the occurrence of dual-phase EPSCs differed among groups, we quantified the proportion of neurons exhibiting dual-phase responses in the first EPSC or in EPSCs two through four of the stimulus train (Supplementary Figure 2). No differences in incidence were detected among experimental groups.

### 3.3 Acute stress alters sexually dimorphic NMDA receptor signaling at IL-to-PH synapse

**Figure 3:**
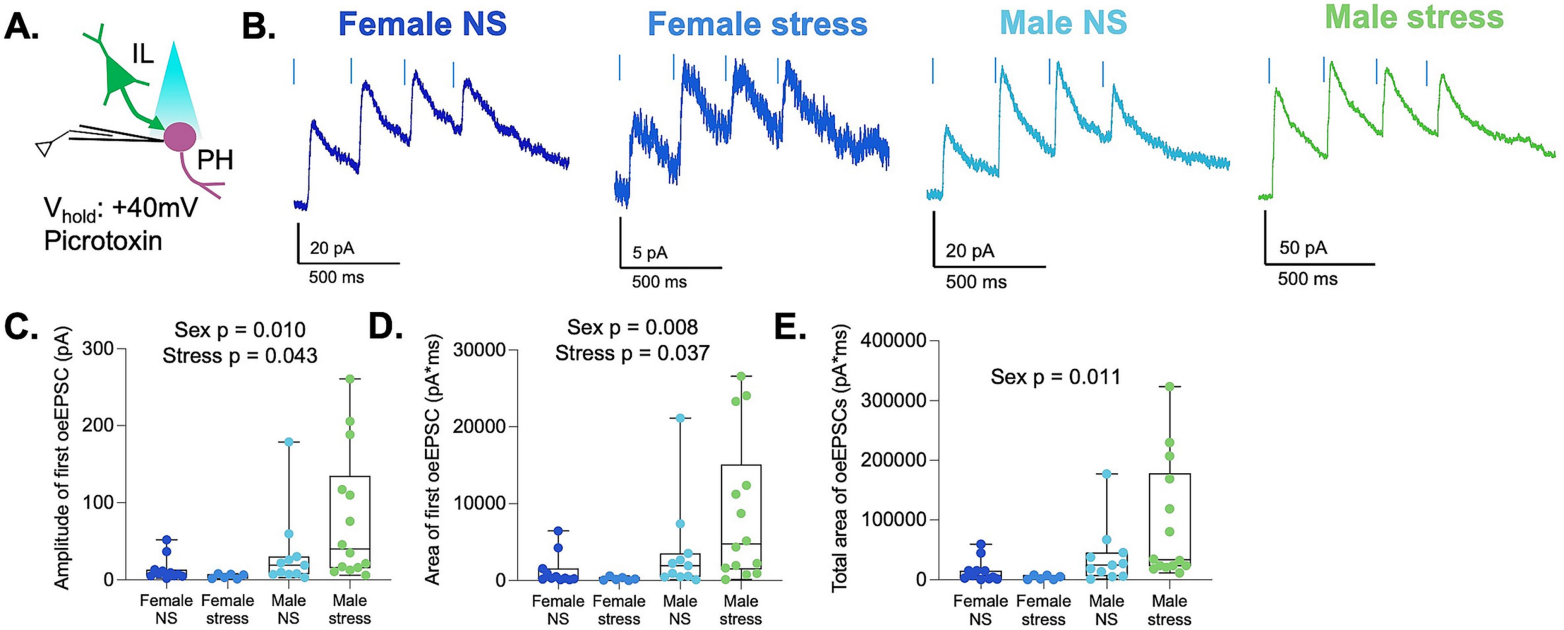
NMDA synaptic responses at IL-to-PH synapses are sexually dimorphic and enhanced by acute stress. **(A)** Schematic depicting optogenetic stimulation of IL terminals and recording of a train of four optically evoked EPSCs at +40 mV in PH neurons. **(B)** Representative traces of optically evoked EPSCs. **(C)** Amplitude, **(D)** area, **(E)** analysis of the total area of optically evoked EPSC across a train of four stimuli in females and male rats. Neurons are the same as in Figure 2. Female NS *n* = 4 rats/11 cells; females stress *n* = 3 rats/6 cells; males NS *n* = 4 rats/11 cells; males stress *n* = 4 rats/14 cells.

Studies have shown that acute stress can induce NMDA receptor depression in the paraventricular nucleus of the hypothalamus of prepubescent male rats (Kuzmiski et al., 2010). However, whether the effects of acute stress on NMDA receptor activity are similar in other hypothalamic regions, such as the PH, is not known. In addition, previous studies have not assessed the effects of acute stress on the activity of NMDA receptors in the PH of female rats or on specific prefrontal inputs. To answer these questions, we examined EPSCs recorded in the same population of PH neurons by optically stimulating IL terminals in the presence of picrotoxin at a holding potential of +40 mV (Figures 3A,B). To isolate NMDAR currents, we analyzed the currents 70–230 ms after light stimulation. A main effect of sex was observed for NMDA EPSC amplitudes [*F* (1,38) = 7.45, *p* = 0.010], total charge transfer of single EPSCs [*F* (1,38) = 7.96, *p* = 0.008], and total charge transfer of EPSC train [*F* (1,38) = 7.17, *p* = 0.011]. This indicates that the synaptic responses at +40 mV were sexually dimorphic, with females showing smaller synaptic NMDAR currents (Figures 3C–E). Collapsing across sex, there was a main effect of stress for NMDA EPSC amplitudes [*F* (1,38) = 4.38, *p* = 0.043] and total charge transfer of single EPSCs [*F* (1,38) = 4.59, *p* = 0.037]. Acute stress was associated with greater EPSC amplitudes overall, although the magnitude of this effect appeared more pronounced in males. There was no main effect of stress on the total charge transfer of EPSC train [*F* (1,38) = 3.31, *p* = 0.077].

### 3.4 Sex modulates EPSC strength at −40 mV

**Figure 4:**
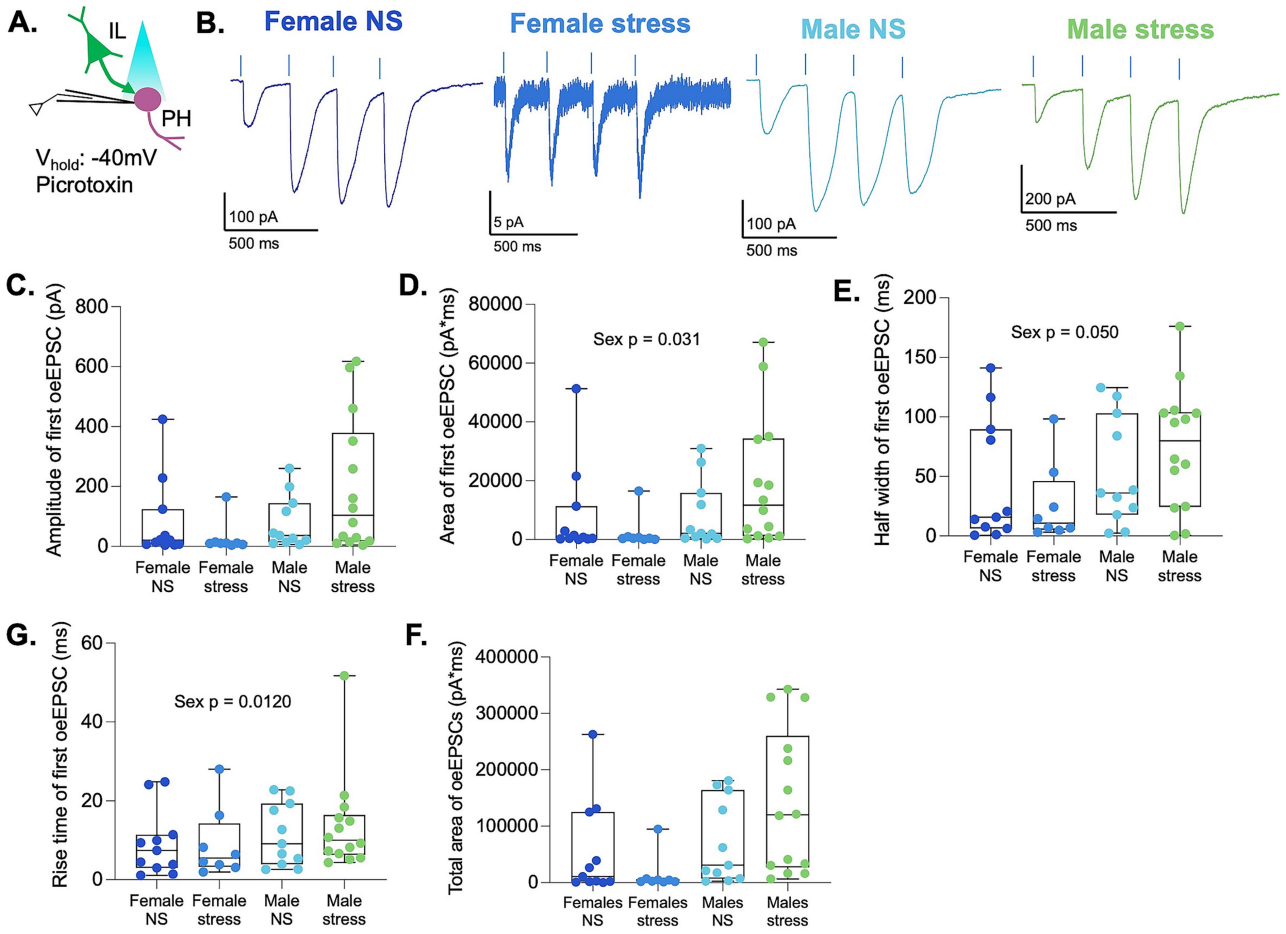
Mixed AMPA/NMDA EPSCs at −40 mV show sexual dimorphism in synaptic charge and kinetics. **(A)** Schematic depicting optogenetic stimulation of IL terminals and recording of optically evoked EPSCs at −40 mV in PH neurons. **(B)** Representative traces of a train of four optically evoked EPSCs in female and male rats. **(C)** Amplitude, **(D)** area, **(E)** half-width, and **(F)** rise time of optically evoked EPSCs recorded at −40 mV in response to the first stimulus. **(G)** Total area of the train of four stimuli in the groups. Neurons are the same as in Figures 2, 3.

To further characterize the postsynaptic responses within the IL-to-PH pathway, we recorded optically evoked EPSCs from the same population of PH neurons while holding the membrane potential at −40 mV in the presence of picrotoxin. At this membrane potential, NMDA receptor activation is greater due to reduced voltage-dependent Mg^2+^ block, allowing greater NMDAR contribution to synaptic currents compared with recordings at −60 mV (Luscher and Malenka, 2012). At a holding potential of −40 mV, we delivered a train of four 1-ms light pulses at 15% light intensity and analyzed the mixed AMPAR/NMDAR EPSCs evoked by the first stimulus (Figures 4A,B). Two-way ART ANOVA revealed no main effects of sex, stress, or interaction on EPSC amplitude or rise time (Figures 4C,F). In contrast, a significant main effect of sex was observed for the total charge transfer of single EPSCs [*F* (1,40) = 5.01, *p* = 0.031] and for EPSC half-width [*F* (1,40) = 4.10, *p* = 0.050], indicating that females exhibited smaller and faster synaptic currents than males (Figures 4D,E). Analysis of the cumulative charge transfer across the four-stimulus train (Figure 4G) similarly revealed a main effect of sex [*F* (1,40) = 6.92, *p* = 0.012], with females showing reduced total current relative to males. No effects of stress or sex × stress interactions were detected for this measure. Together, these findings indicate that glutamatergic synaptic responses recorded at −40 mV exhibit sexual dimorphism in synaptic charge and kinetics, whereas acute restraint stress does not alter these postsynaptic properties in the IL-to-PH pathway.

### 3.5 Sex and stress shape NMDAR-predominant spontaneous synaptic inputs to PH neurons

**Figure 5:**
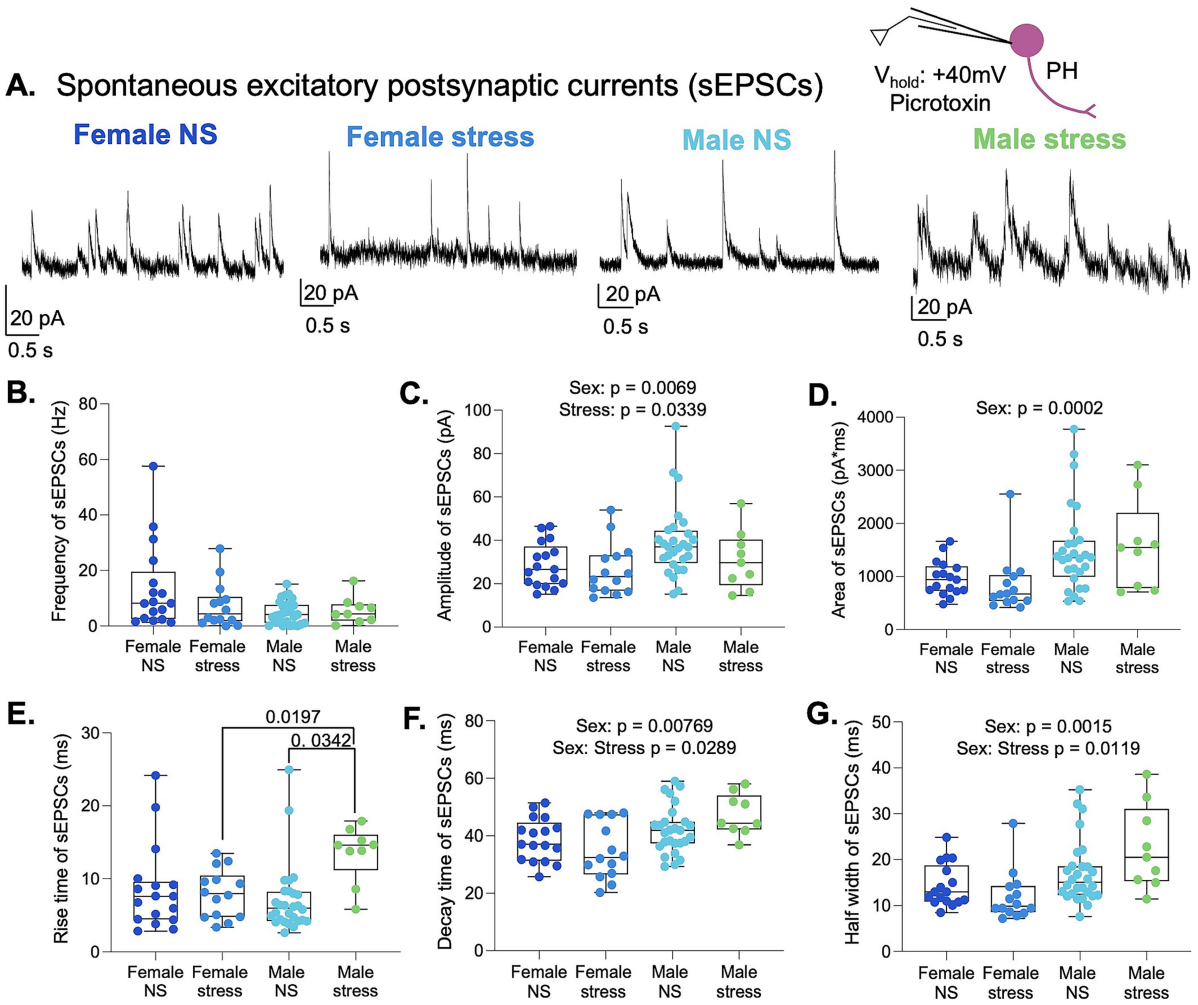
Sex and stress shape NMDAR-predominant spontaneous synaptic inputs to PH neurons. **(A)** Representative traces of sEPSCs and a schematic depicting the recording of sEPSCs at +40 mV with PTX in PH neurons. **(B)** Frequency, **(C)** amplitude, **(D)** area, **(E)** rise time, **(F)** decay time, and **(G)** half-width of sEPSCs. Female NS *n* = 5 rats/17 cells, Female stress *n* = 4 rats/14 cells, Male NS *n* = 9 rats/28 cells, Male stress *n* = 3 rats/9 cells.

The PH receives innervation from several brain regions involved in stress responses, including the thalamus and the amygdala (Çavdar et al., 2001). To determine whether the observed effects of sex and acute stress were specific to the IL-to-PH pathway or impacted most synaptic inputs to PH neurons, we examined NMDAR-predominant sEPSCs in the PH of male and female rats. Male and female rats were restrained for 30 min. Then, acute PH brain slices were prepared to measure sEPSCs at a holding potential of +40 mV in the presence of picrotoxin to isolate excitatory currents under conditions that favor NMDAR activation (Figure 5A). Because the data did not pass normality testing, we used an ART ANOVA test to compare the frequency, amplitude, total charge transfer, rise time, decay time, and half-width of sEPSCs for the effects of sex, stress, and interactions. No differences in sEPSC frequency were detected, suggesting that males and females receive similar numbers of synaptic inputs that are not altered by acute stress (Figure 5B). The amplitude [*F* (1,64) = 7.80, *p* = 0.0069] and total charge transfer [*F* (1,64) = 15.77, *p* = 0.0002] showed a main effect of sex (Figures 5C, D), suggesting that females have smaller NMDAR-predominant excitatory synaptic inputs to PH neurons. Collapsing across sex, the amplitude was also altered by stress exposure [*F* (1,64) = 4.70, *p* = 0.034], with smaller amplitudes after stress exposure. The kinetics of the sEPSCs were also affected by sex and stress (Figures 5E–G). We detected a main effect of stress [*F* (1,64) = 12.04, *p* = 0.0009] and a significant sex × stress interaction [*F* (1,64) = 9.95, *p* = 0.0025] in the sEPSC rise time. *Post hoc* pairwise comparisons indicated that stressed males exhibited longer rise times compared with non-stressed males (*p* = 0.0342) and stressed females (*p* = 0.0197). Non-stressed males exhibited shorter rise times than stressed males, suggesting that acute stress may slow the kinetics of postsynaptic responses in PH neurons of male rats. The decay time showed a main effect of sex [*F* (1,63) = 7.58, *p* = 0.0077] and a significant interaction [F (1,63) = 5.00, *p* = 0.0289], with faster decay kinetics observed in females. However, *post hoc* pairwise comparisons did not reach statistical significance. Finally, sEPSC half-width exhibited a main effect of sex [*F* (1,64) = 11.08, *p* = 0.0015] and a significant interaction [*F* (1,64) = 6.70, *p* = 0.0119], with females showing smaller half-widths. Overall, these data demonstrate that sex and acute stress shape the strength and kinetics of NMDAR-predominant spontaneous excitatory synaptic currents in PH neurons, revealing broader modulation of excitatory signaling within the PH.

### 3.6 Intact GABAergic inhibition occludes effects of sex on IL-to-PH synaptic transmission

**Figure 6:**
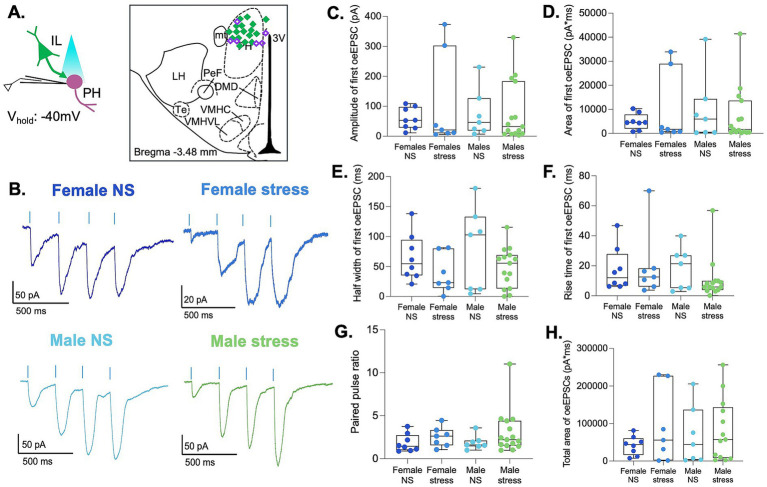
GABAergic inhibition occludes the effects of sex on IL-to-PH synaptic transmission. **(A)** Schematic depicting optogenetic stimulation of IL terminals and recording of optically evoked EPSCs at −40 mV in PH neurons without PTX. **(B)** Representative traces of optically evoked EPSCs from each group. **(C)** Amplitude, **(D)** area, **(E)** half-width, **(F)** rise time, and **(G)** PPR of optically evoked EPSCs across groups. **(H)** Analysis of the total area of optically evoked EPSC across a train of four stimuli in females and male rats. Female NS *n* = 3 rats/8 cell. Female stress *n* = 3 rats/7 cells. Male NS *n* = 3 rats/7 cells. Male stress *n* = 4 rats/15 cells.

Based on histological evidence that IL glutamatergic neurons innervate GABAergic neurons in the PH, researchers propose that IL activation primarily engages PH GABAergic neurons to suppress PH activity (Myers et al., 2016). Therefore, optogenetic stimulation of IL terminals may recruit local inhibitory circuits within the PH. To test this hypothesis, we recorded optically evoked EPSCs in the PH at −40 mV in the absence of picrotoxin to examine whether feedforward inhibition is engaged by IL-to-PH pathway activation (Figure 6A). Under these recording conditions, the difference in the Nernst potential for Na^+^ and Cl^−^ results in inward EPSCs and outward inhibitory postsynaptic currents (IPSCs), enabling detection of both excitatory and inhibitory components of the synaptic response. Across 35 synaptic connections examined, we found no evidence of feedforward inhibition, such as biphasic responses or delayed IPSCs (Figure 6B). Consistent with the recordings with GABAergic transmission blocked with picrotoxin, recordings with intact GABAergic inhibition revealed no main effects of sex or stress on the first EPSC amplitude (Figure 6C), rise time (Figure 6F), or PPR (Figure 6G). In contrast to the previous results with picrotoxin, no effect of sex was detected on the total charge transfer of a single EPSC (Figure 6D), EPSC half-width (Figure 6E), or the total charge transfer of four EPSCs (Figure 6H). Taken together, these findings suggest that local inhibitory tone may buffer the effects of sex on excitatory synaptic transmission.

Because recordings at −40 mV permit a substantial NMDAR-mediated current, we considered the possibility that prolonged inward NMDA currents could obscure outward feedforward IPSCs evoked by optical stimulation of glutamatergic terminals from the IL. To evaluate whether overlapping inhibitory currents influenced the measured EPSCs, we compared responses recorded in the presence and absence of GABA_A_ receptor blockade. If feedforward IPSCs were present but masked by the NMDA component, removal of GABA_A_ receptor transmission would be expected to increase inward peak amplitude, increase charge transfer, or broaden the EPSC waveform.

We therefore compared synaptic responses obtained with and without picrotoxin. In males, blockade of GABA_A_ receptors did not alter the amplitude, charge transfer, or half-width of the first EPSC, nor the total charge transfer of the train of four EPSCs (Supplementary Figures 3A–D). In females, the peak amplitude (Supplementary Figure 3E) and half-width (Supplementary Figure 3G) of the first EPSC were also unchanged; however, the charge transfer of the first EPSC (Supplementary Figure 3F) and the total charge transfer across the four-pulse train (Supplementary Figure 3H) were reduced in the presence of picrotoxin. Together, these results indicate that GABA_A_ receptor blockade did not produce the expected increases in inward current or waveform broadening if outward IPSCs were masking EPSCs, suggesting that the recorded responses are unlikely to reflect truncated excitatory currents caused by overlapping inhibitory inputs. While our data did not reveal detectable feedforward inhibitory currents under the conditions tested, we cannot exclude the possibility that weak temporally overlapping inhibition may be present but remained below our detection threshold.

We also considered the possibility that tonic GABA_A_-mediated inhibition could occlude the observed effects by shunting synaptic currents. To evaluate this possibility, we compared baseline holding current and input resistance (Rm) between neurons recorded in the presence or absence of picrotoxin. If tonic GABA_A_ conductance was substantial, blocking GABA_A_ receptors would be expected to reduce outward holding current and increase input resistance. In females, holding current and input resistance did not differ between recordings obtained with or without picrotoxin. In males, picrotoxin was associated with larger baseline holding currents and lower input resistance (Supplementary Figures 3I–L). These findings do not support the presence of a substantial tonic inhibitory conductance under our recording conditions and therefore argue against significant shunting of synaptic currents by tonic GABA_A_ activity.

### 3.7 Lack of effects of sex or acute stress on sEPSC activity in the PH with intact GABAergic inhibition

**Figure 7:**
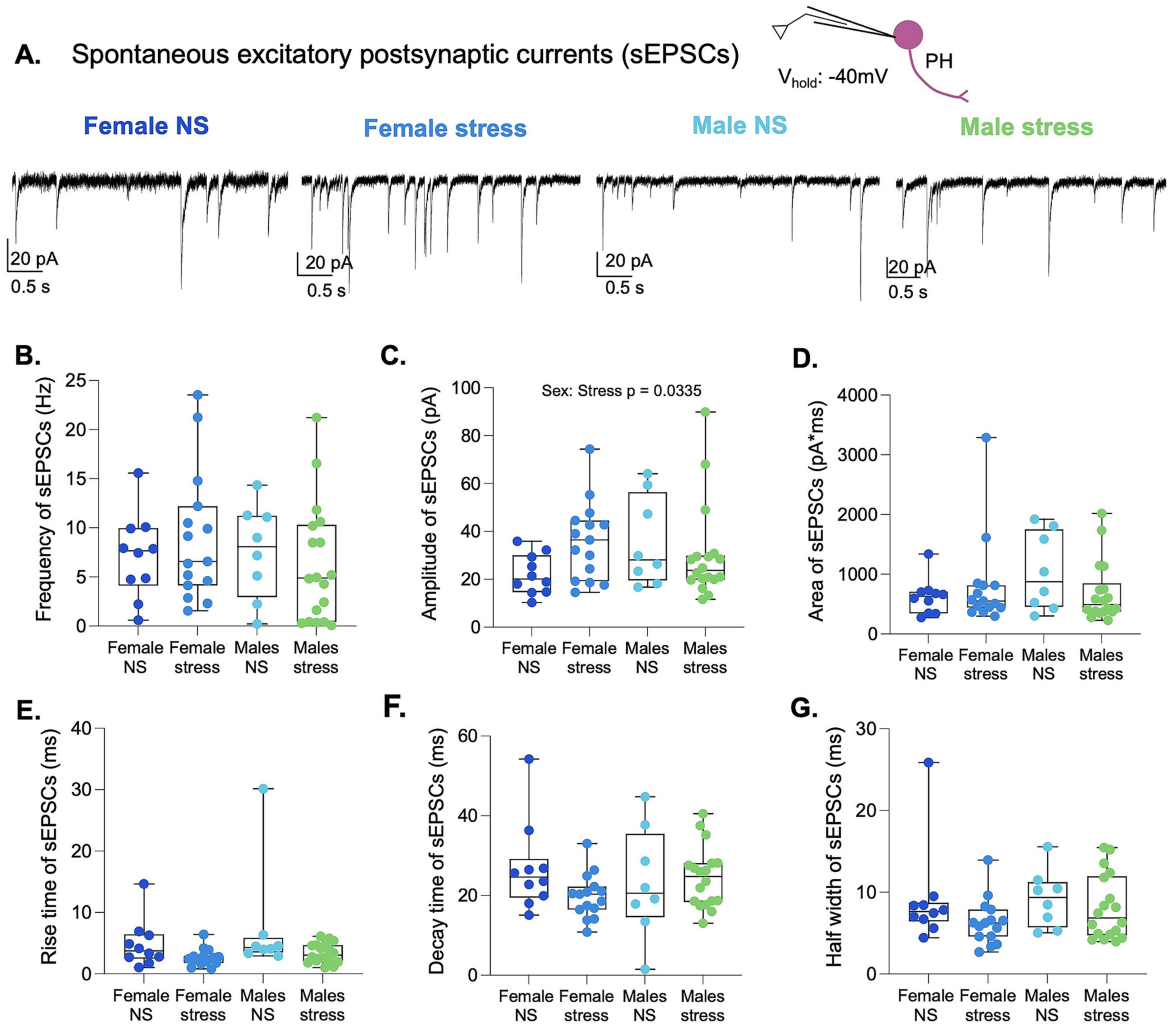
Lack of effects of sex or acute stress on sEPSC activity in the PH with intact GABAergic inhibition. **(A)** Representative traces of sEPSCs and a schematic depicting whole-cell patch-clamp recording of sEPSCs at −40 mV without PTX in PH neurons. **(B)** Frequency, **(C)** amplitude, **(D)** area, **(E)** rise time, **(F)** decay time, and **(G)** half-width of sEPSCs. Female NS *n* = 4 rats/10 cells. Female stress *n* = 4 rats/15 cells. Male NS *n* = 3 rats/8 cells. Male stress *n* = 4 rats/18 cells.

To further examine the effects of acute stress on sEPSCs in PH neurons with intact GABAergic inhibition, animals were restrained for 30 min, after which we prepared acute PH brain slices for whole-cell patch-clamp recordings at a holding potential of −40 mV (Figure 7A). Since the data did not meet assumptions of normality, we used the ART ANOVA test to compare the frequency, amplitude, total charge transfer, rise time, decay time, and half-width of sEPSCs for main effects of stress, sex, or interaction (Figures 7B–G). The analysis did not reveal a main effect of stress or sex in any of the measurements. An interaction was detected for amplitude (F (1,47) = 4.80, *p* = 0.0335). *Post hoc* pairwise comparisons were not significant. Together, these findings further suggest that local inhibitory tone may buffer the effects of sex or acute stress on glutamatergic input to PH neurons in both sexes.

## 4 Discussion

### 4.1 Functional sexual dimorphism in the IL-to-PH pathway

Our findings reveal that single or burst stimulation of glutamatergic inputs from the IL evoked smaller responses in PH neurons of females at all holding potentials. Similar sex differences were evident in NMDAR-predominant sEPSCs at +40 mV, with females exhibiting smaller and faster currents. When data were collapsed across sex, acute restraint stress was associated with increased amplitude and total charge of optically evoked NMDAR-mediated EPSCs in the IL-to-PH pathway and a reduction in spontaneous NMDAR-predominant EPSCs. In contrast, acute stress had little effect on mixed glutamatergic responses at −60 or −40 mV. Together with the unchanged PPR or frequency of sEPSCs, these results suggest that the effects of acute stress arise from postsynaptic receptor function or channel gating, rather than presynaptic release probability. These intrinsic differences suggest that sex strongly shapes prefrontal-hypothalamic communication (Herman et al., 2020; Myers et al., 2016; Schaeuble et al., 2024), and acute stress enhances the IL synaptic signaling while depressing signaling from other structures. Our data is consistent with *in vivo* observations showing that optogenetic stimulation of IL terminals in the PH during stress increases corticosterone levels in females but not males (Schaeuble et al., 2024), demonstrating that the IL-to-PH circuit is functionally sexually dimorphic.

Functional sexual dimorphism has been identified in other nodes of stress-integrative circuitry. For example, in the posterior paraventricular thalamic nucleus, males exhibit greater sEPSC enhancement during acute restraint stress than females (Corbett et al., 2022). Although our study did not find sex-dependent effects of acute stress on IL-to-PH synaptic strength, the baseline sex differences we observed and the sex-dependent endocrine responses described in the literature (Schaeuble et al., 2024) suggest that male and female stress circuits differ in their intrinsic excitatory integration and output patterns, even when acute excitatory synaptic plasticity is not sex-dependent. In this context, the PH may rely more on sexually differentiated baseline excitatory properties rather than on sex-specific amplitude changes in synaptic strength. Such functional dimorphism across interconnected stress-regulatory regions may contribute to known sex differences in stress coping and vulnerability.

### 4.2 Sex and stress modulate NMDAR signaling in the PH

Sex differences were evident in the baseline properties of NMDAR-mediated currents within the IL-to-PH pathway. In optically evoked responses, female neurons exhibited smaller NMDA-mediated EPSCs than males, suggesting intrinsic differences in receptor composition or gating at IL-to-PH synapses. These baseline differences may place female PH neurons in a different functional operating range than males, potentially influencing how excitatory signals arriving from the IL are integrated within this pathway. When NMDAR-predominant sEPSCs were examined in PH neurons, additional sex-dependent differences were observed in the kinetics of these events, with females displaying faster decay properties. Together, these observations suggest that sex influences both the strength of NMDAR-mediated transmission at IL-to-PH synapses and the temporal properties of spontaneous excitatory inputs to PH neurons.

Acute stress enhanced NMDAR-mediated synaptic responses at IL-to-PH synapses. Optically evoked EPSCs recorded at +40 mV showed increased NMDA receptor-mediated amplitudes and greater charge transfer following stress exposure, indicating that acute stress strengthens NMDAR signaling within this prefrontal-hypothalamic pathway. This effect was observed when data were collapsed across sex, although the magnitude of the increase appeared greater in males, suggesting that baseline sex differences in NMDAR function may influence how this pathway responds to stress. These findings indicate that acute stress can potentiate NMDA-dependent excitatory transmission from the IL-to-PH. In addition, acute stress also altered NMDAR-predominant spontaneous synaptic currents in PH neurons, indicating that stress influences excitatory signaling in the PH beyond specific long-range pathways. However, in contrast to the IL-to-PH optogenetic recordings, spontaneous NMDAR-predominant events recorded in PH neurons showed reduced amplitudes following stress exposure. Consistent with our findings, stress alters NMDAR signaling in other hypothalamic regions (Bains et al., 2015; Zhou et al., 2018) and may reflect variation in receptor subunit composition or channel gating that shapes synaptic integration. In summary, acute stress enhances NMDAR-mediated signaling at IL-to-PH synapses, while reducing overall NMDAR signaling in the PH. This suggests that acute stress may preferentially increase the impact of prefrontal inputs on hypothalamic activity during stressful conditions.

### 4.3 IL burst encoding and amplification of subcortical synaptic drive

The short-term facilitation observed at IL-to-PH synapses is consistent with synaptic dynamics reported in other IL-associated circuits. For instance, in male rats, IL excitatory inputs to principal neurons in the basolateral amygdala also exhibit short-term facilitation, which is further enhanced following fear extinction training (Cho et al., 2013). Facilitating synapses are characterized by their ability to amplify postsynaptic responses during bursts of presynaptic activity, consistent with a role in amplifying postsynaptic responses during temporally clustered presynaptic activity. Notably, burst firing of IL neurons is necessary for the consolidation of fear extinction memory (Burgos-Robles et al., 2007). These findings suggest that bursts of IL activity may serve as a general mechanism to enhance synaptic drive onto subcortical targets, including the PH, particularly under conditions of emotional arousal or stress. The facilitation we observed at IL-to-PH synapses may therefore reflect a conserved feature of IL output pathways that support adaptive behavioral and physiological responses to stress.

### 4.4 Inhibitory tone and the role of feedforward inhibition

Given that IL glutamatergic neurons are known to synapse onto GABAergic neurons in the PH (Myers et al., 2016), we explored the possibility of feedforward inhibition by recording EPSCs at −40 mV without GABA receptor blockade. Under these conditions, we did not observe clear evidence of feedforward inhibition, such as biphasic responses or delayed inhibitory postsynaptic currents. Interestingly, the sex-dependent differences in optically evoked EPSCs observed under GABA blockade were no longer present when inhibition was intact. This suggests that local inhibitory tone may buffer or mask excitatory differences. However, blockade of GABA_A_ receptors in slice preparations can remove inhibitory constraints within the local network, potentially increasing excitatory neurotransmitter or neuromodulator release and activating additional membrane conductances that could alter membrane resistance and influence EPSC kinetics. Consistent with this possibility, neurons recorded in the presence of picrotoxin exhibited larger baseline holding currents and reduced input resistance in males, indicating that GABA_A_ receptor blockade may engage additional network or intrinsic conductances under these conditions. Thus, while our results suggest that intact inhibitory signaling may constrain the expression of sex-dependent differences in excitatory synaptic responses in the PH (Herman et al., 2020; Myers et al., 2016), network effects associated with GABA_A_ receptor blockade represent a potential limitation when comparing synaptic responses across these conditions.

### 4.5 Functional implications for stress adaptation and sex differences

Taken together, our data position the PH as a sex-modulated integrative hub in which baseline excitatory signaling differs between males and females, and acute stress tunes the strength of NMDAR signaling. These findings complement evidence that the IL-to-PH pathway contributes to autonomic and neuroendocrine stress responses (Herman et al., 2020; Schaeuble et al., 2024) and underscore the importance of including sex as a biological variable in circuit-level studies of stress.

Future work should determine the molecular determinants of these changes (e.g., NMDAR subunit composition, Mg^2+^ block sensitivity, or AMPAR auxiliary proteins) and use cell-type-specific approaches to identify whether specific PH neuronal populations (glutamatergic vs. GABAergic) show distinct synaptic modulation. Finally, *in vivo* manipulations that preserve inhibitory tone will be essential to assess how GABAergic buffering governs prefrontal control of hypothalamic stress output under acute stress.

### 4.6 Strengths and limitations

This study provides the first comprehensive characterization of IL-to-PH synaptic physiology in both male and female rats, including how this pathway responds to acute restraint stress. A major strength of our approach is the use of optogenetics to selectively activate IL inputs, enabling precise interrogation of a defined prefrontal-hypothalamic circuit. Our electrophysiological design allowed us to assess both evoked and spontaneous excitatory synaptic currents across multiple holding potentials, providing a detailed profile of baseline sex differences and stress-induced kinetic modulation within PH neurons.

Several limitations should be acknowledged. First, we did not pharmacologically isolate AMPA receptor currents, which limits our ability to definitively attribute changes at −60 mV and −40 mV to AMPAR or NMDAR. Second, we did not identify the specific PH neuronal subtypes recorded. Although the PH is predominantly glutamatergic, it also contains GABAergic neurons and neurons expressing hypocretin, tyrosine hydroxylase, neuropeptide Y, and serotonin (Abrahamson and Moore, 2001). It is therefore possible that some recordings were obtained from GABAergic neurons, which may explain the absence of detectable feedforward inhibition. Third, our acute stress paradigm may not capture the full range of stress-induced plasticity occurring with different intensities, durations, or modalities of stressors. Future studies incorporating cell-type-specific labeling, receptor-selective pharmacology, and alternative stress models will be important for identifying the cellular substrates underlying sex differences in baseline excitatory signaling and stress-induced kinetic changes.

## 5 Conclusion

Taken together, our findings indicate that prefrontal-hypothalamic communication is shaped by both baseline sex differences and acute stress-dependent modulation of NMDAR signaling. Female PH neurons exhibited smaller NMDAR-mediated synaptic responses at IL-to-PH synapses, revealing a functional sexual dimorphism in this pathway. Acute restraint stress enhanced NMDAR-mediated synaptic responses evoked by IL inputs while simultaneously reducing the amplitude of spontaneous NMDAR-predominant excitatory currents recorded in PH neurons. These results suggest that acute stress differentially modulates excitatory signaling within the PH, strengthening prefrontal cortical inputs while dampening broader excitatory drive from other afferent sources. Such selective modulation may increase the relative influence of prefrontal control over hypothalamic activity during stressful conditions.

These results provide new insight into how prefrontal regulation of hypothalamic circuits may be adapted during stress. Our findings emphasize that sex is a critical biological variable that shapes baseline excitatory architecture, while acute stress modifies the information flow within hypothalamic stress-regulatory circuits.

## Data Availability

The raw data supporting the conclusions of this article will be made available by the authors, without undue reservation.
