# Supplementary material for: Sexual dimorphism and acute stress modulation of infralimbic-posterior hypothalamic synaptic transmission
Source: Front Cell Neurosci. 2026 Apr 10;20:1659293. doi: 10.3389/fncel.2026.1659293 (PMC13105900; doi:10.3389/fncel.2026.1659293)
Supplement: Supplementary file 1 [file Table_1.docx]

| Statistical table | | | |
| --- | --- | --- | --- |
| Figure | **Data structure** | **Mean (SD)** | **ART ANOVA** |
| Fig. 2D | Non-parametric | Female NS: 101.24 ± 135.66  Female stress: 32.42 ± 47.84  Male NS: 80.58 ± 67.52  Male stress: 211.94 ± 260.71 | Sex: F (1,40) 2.12, p = 0.153  Stress: F (1,40) 0.30, p = 0.586  Sex: Stress: F (1,40) 1.68, p = 0.202 |
| Fig. 2E | Non-parametric | Female NS: 6627.8 ± 11504.07  Female stress: 2417.52 ± 5206.42  Male NS: 6424.07 ± 9032.26  Male stress: 11940.87 ± 16005.5 | Sex: F (1,40) 4.10, p = 0.0495  Stress: F (1,40) 0.56, p = 0.4590  Sex: Stress: F (1,40) 2.25, p = 0.1420 |
| Fig. 2F | Non-parametric | Female NS: 24.23 ± 30.37  Female stress: 28.33 ± 28.51  Male NS: 36.73 ± 46.11  Male stress: 30.03 ± 19.13 | Sex: F (1,40) 1.30, p = 0.261  Stress: F (1,40) 1.22, p = 0.277  Sex: Stress: F (1,40) 0.06, p = 0.815 |
| Fig. 2G | Non-parametric | Female NS: 4.41 ± 2.08  Female stress: 9.95 ± 7.54  Male NS: 7.69 ± 8.71  Male stress: 5.9 ± 3.03 | Sex: F (1,39) 0.36, p = 0.551  Stress: F (1,39) 1.26, p = 0.268  Sex: Stress: F (1,39) 1.12, p = 0.296 |
| Fig. 2H | Non-parametric | Female NS: 1.66 ± 0.8  Female stress: 1.31 ± 0.38  Male NS: 1.55 ± 0.59  Male stress: 1.51 ± 0.4 | Sex: F (1,40) 0.03, p = 0.858  Stress: F (1,40) 0.43, p = 0.518  Sex: Stress: F (1,40) 0.68, p = 0.414 |
| Fig. 2I | Non-parametric | Female NS: 47074.12 ± 73339.71  Female stress: 14017.33 ± 29114.67  Male NS: 48508.71 ± 62742.61  Male stress: 99440.41 ± 103028.28 | Sex: F (1,40) 5.58, p = 0.0231  Stress: F (1,40) 1.22, p = 0.2760  Sex: Stress: F (1,40) 3.88, p = 0.0559 |
| Fig. 3C | Non-parametric | Female NS: 13.99 ± 15.7  Female stress: 4.5 ± 2.98  Male NS: 33.61 ± 50.79  Male stress: 80.04 ± 84.15 | Sex: F (1,38) 7.45, p = 0.00955  Stress: F (1,38) 4.38, p = 0.04310  Sex: Stress: F (1,38) 3.22, p = 0.08050 |
| Fig. 3D | Non-parametric | Female NS: 1369.64 ± 2077.94  Female stress: 240.3 ± 226.74  Male NS: 3771.84 ± 6120.82  Male stress: 8825.85 ± 9423.53 | Sex: F (1,38) 7.96, p = 0.00758  Stress: F (1,38) 4.59, p = 0.03860  Sex: Stress: F (1,38) 3.22, p = 0.08080 |
| Fig. 3E | Non-parametric | Female NS: 15207.71 ± 19419.23  Female stress: 3827.32 ± 3512.66  Male NS: 38505.81 ± 50048.35  Male stress: 94088.21 ± 100209.18 | Sex: F (1,38) 7.17, p = 0.0109  Stress: F (1,38) 3.31, p = 0.0768  Sex: Stress: F (1,38) 3.05, p = 0.0891  Post-hoc pairwise comparison (Tukey-adjusted) Female NS-Male NS: p = 0.10529103, Female NS-Female stress: p = 0.08954787, Female NS-Male stress: p = 0.04509884, Male NS - Female Stress: p = 0.96879159, Male NS - Male Stress: p = 0.99461752, Female Stress - Male Stress: p = 0.99326894 |
| Fig. 3F | Non-parametric | Female NS: 6.28 ± 5.79  Female stress: 6.76 ± 5.99  Male NS: 3.61 ± 2.22  Male stress: 2.68 ± 2.55 | Sex: F (1,38) 3.61, p = 0.0651  Stress: F (1,38) 1.52, p = 0.2250  Sex: Stress: F (1,38) 0.23, p = 0.6340 |
| Fig. 4C | Non-parametric | Female NS: 81.36 ± 133.34  Female stress: 29.03 ± 55.11  Male NS: 79.78 ± 87.52  Male stress: 197.42 ± 222.96 | Sex: F (1,40) 3.35, p = 0.0748  Stress: F (1,40) 0.99, p = 0.3250  Sex: Stress: F (1,40) 2.46, p = 0.1250 |
| Fig. 4D | Non-parametric | Female NS: 8253.75 ± 15771.87  Female stress: 2451.98 ± 5680.5  Male NS: 8603.31 ± 11187.48  Male stress: 19128.44 ± 21960.38 | Sex: F (1,40) 5.01, p = 0.0308  Stress: F (1,40) 0.95, p = 0.3360  Sex: Stress: F (1,40) 2.45, p = 0.1250 |
| Fig. 4E | Non-parametric | Female NS: 44.85 ± 51.76  Female stress: 26.49 ± 33.39  Male NS: 53.02 ± 45.64  Male stress: 74.73 ± 51.17 | Sex: F (1,40) 4.10, p = 0.0495  Stress: F (1,40) 1.18, p = 0.2850  Sex: Stress: F (1,40) 2.32, p = 0.1360. |
| Fig. 4F | Non-parametric | Female NS: 9.19 ± 8.3  Female stress: 9.05 ± 8.88  Male NS: 11.4 ± 7.95  Male stress: 13.75 ± 12.13 | Sex: F (1,40) 2.69, p = 0.109  Stress: F (1,40) 0.43, p = 0.515  Sex: Stress: F (1,40) 0.13, p = 0.718 |
| Fig. 4G | Non-parametric | Female NS: 54879.62 ± 84369.26  Female stress: 14764.89 ± 32369.53  Male NS: 72002.7 ± 74105.76  Male stress: 143046.06 ± 127174.15 | Sex: F (1,40) 6.92, p = 0.0120  Stress: F (1,40) 1.16, p = 0.2890  Sex: Stress: F (1,40) 3.48, p = 0.0694 |
| Fig. 5B | Non-parametric | Female NS: 13.72 ± 15.28  Female stress: 7.39 ± 8.11  Male NS: 4.88 ± 4.17  Male stress: 5.64 ± 4.81 | Sex: F (1,64) 3.49, p = 0.0665  Stress: F (1,64) 0.15, p = 0.7010  Sex: Stress: F (1,64) 1.63, p = 0.2060 |
| Fig. 5C | Non-parametric | Female NS: 28.61 ± 10.14  Female stress: 26.54 ± 12.07  Male NS: 39.24 ± 16.45  Male stress: 30.97 ± 13.57 | Sex: F (1,64) 7.80, p = 0.00687  Stress: F (1,64) 4.70, p = 0.03390  Sex: Stress: F (1,64) 0.65, p = 0.42300 |
| Fig. 5D | Non-parametric | Female NS: 967.02 ± 327.72  Female stress: 846.13 ± 540.47  Male NS: 1507.63 ± 813.53  Male stress: 1598.56 ± 844.41 | Sex: F (1,64) 15.77, p = 0.000184  Stress: F (1,64) 2.55, p = 0.115000  Sex: Stress: F (1,64) 0.95, p = 0.334000 |
| Fig. 5E | Non-parametric | Female NS: 8.63 ± 5.84  Female stress: 7.99 ± 3.31  Male NS: 7.14 ± 4.76  Male stress: 13.48 ± 3.86 | Sex: F (1,64) 0.86, p = 0.357000  Stress: F (1,64) 12.04, p = 0.000936  Sex: Stress: F (1,64) 9.95, p = 0.002450. Post-hoc pairwise comparison (Tukey-adjusted) Female NS-Male NS: p = 0.60689857, Female NS-Female stress: p = 0.36414198, Female NS-Male stress: p = 0.34727243, Male NS - Female Stress: p = 0.91611488, Male NS - Male Stress: p = 0.03423508, Female Stress - Male Stress: p = 0.01974764 |
| Fig. 5F | Non-parametric | Female NS: 38.61 ± 7.48  Female stress: 34.85 ± 9.85  Male NS: 42.11 ± 8.2  Male stress: 47.41 ± 7.15 | Sex: F (1,63) 7.58, p = 0.00769  Stress: F (1,63) 0.32, p = 0.57100  Sex: Stress: F (1,63) 5.00, p = 0.02890. Post-hoc pairwise comparison (Tukey-adjusted) Female NS-Male NS: p = 0.6313773, Female NS-Female stress: p = 0.7281914, Female NS-Male stress: p = 0.7178962, Male NS - Female Stress: p = 0.9999993, Male NS - Male Stress: p = 0.1668281, Female Stress - Male Stress: p = 0.2409087 |
| Fig. 5G | Non-parametric | Female NS: 14.35 ± 4.62  Female stress: 12.02 ± 5.4  Male NS: 17.17 ± 6.9  Male stress: 22.64 ± 9.16 | Sex: F (1,64) 11.08, p = 0.00145  Stress: F (1,64) 0.02, p = 0.89700  Sex: Stress: F (1,64) 6.70, p = 0.01190. Post-hoc pairwise comparison (Tukey-adjusted) Female NS-Male NS: p = 0.1256303, Female NS-Female stress: p = 0.3823371, Female NS-Male stress: p = 0.9954980, Male NS - Female Stress: p = 0.9879975, Male NS - Male Stress: p = 0.1837476, Female Stress - Male Stress: p = 0.3961138 |
| Fig. 6C | Non-parametric | Female NS: 59.51 ± 36.2  Female stress: 108.82 ± 158.23  Male NS: 74.36 ± 79.75  Male stress: 78.66 ± 100.06 | Sex: F (1,33) 0.04, p = 0.836  Stress: F (1,33) 0.91, p = 0.348  Sex: Stress: F (1,33) 0.06, p = 0.809 |
| Fig. 6D | Non-parametric | Female NS: 4943.3 ± 3312.66  Female stress: 9869.94 ± 14805.46  Male NS: 9763.51 ± 13942.41  Male stress: 7343.41 ± 11233.74 | Sex: F (1,33) 0.00, p = 0.966  Stress: F (1,33) 0.54, p = 0.466  Sex: Stress: F (1,33) 0.12, p = 0.732 |
| Fig. 6E | Non-parametric | Female NS: 65.05 ± 39.11  Female stress: 37.6 ± 31.92  Male NS: 79.16 ± 69.54  Male stress: 47.81 ± 33.49 | Sex: F (1,33) 0.06, p = 0.807  Stress: F (1,33) 2.05, p = 0.161  Sex: Stress: F (1,33) 0.02, p = 0.883 |
| Fig. 6F | Non-parametric | Female NS: 28.67 ± 34.75  Female stress: 19.65 ± 22.78  Male NS: 18.36 ± 14.13  Male stress: 10.43 ± 13.68 | Sex: F (1,33) 1.50, p = 0.229  Stress: F (1,33) 1.89, p = 0.178  Sex: Stress: F (1,33) 0.03, p = 0.870 |
| Fig. 6G | Non-parametric | Female NS: 1.84 ± 1.04  Female stress: 2.53 ± 1.15  Male NS: 1.89 ± 0.83  Male stress: 3.05 ± 2.53 | Sex: F (1,33) 0.53, p = 0.4700  Stress: F (1,33) 3.57, p = 0.0676  Sex: Stress: F (1,33) 0.02, p = 0.8900 |
| Fig. 6H | Non-parametric | Female NS: 41371.52 ± 25129.99  Female stress: 90346.62 ± 98801.6  Male NS: 67989.82 ± 77853.44  Male stress: 82098.88 ± 82505.03 | Sex: F (1,31) 0.05, p = 0.820  Stress: F (1,31) 0.57, p = 0.454  Sex: Stress: F (1,31) 0.03, p = 0.871 |
| Fig. 7B | Non-parametric | Female NS: 7.12 ± 4.3  Female stress: 8.99 ± 6.63  Male NS: 7.56 ± 4.83  Male stress: 6.23 ± 6.07 | Sex: F (1,47)1.17, p = 0.286  Stress: F (1,47) 0.12, p = 0.736  Sex: Stress: F (1,47) 0.98, p = 0.328 |
| Fig. 7C | Non-parametric | Female NS: 22.06 ± 8.39  Female stress: 35.98 ± 16.31  Male NS: 35.68 ± 18.67  Male stress: 30.22 ± 19.94 | Sex: F (1,47) 0.30, p = 0.5850  Stress: F (1,47) 0.64, p = 0.4270  Sex: Stress: F (1,47) 4.80, p = 0.0335. Post-hoc pairwise comparison (Tukey-adjusted) Female NS-Male NS: p = 0.5364016, Female NS-Female stress: p = 0.6814476, Female NS-Male stress: p = 0.9600292, Male NS - Female Stress: p = 0.9744640, Male NS - Male Stress: p = 0.2155341, Female Stress - Male Stress: p = 0.2586804 |
| Fig. 7D | Non-parametric | Female NS: 623.1 ± 300.91  Female stress: 806.25 ± 758.6  Male NS: 1041.32 ± 649.52  Male stress: 694.75 ± 503.9 | Sex: F (1,47) 0.13, p = 0.720  Stress: F (1,47)1.69, p = 0.200  Sex: Stress: F (1,47) 1.89, p = 0.175 |
| Fig. 7E | Non-parametric | Female NS: 4.86 ± 3.92  Female stress: 2.67 ± 1.39  Male NS: 7.48 ± 9.21  Male stress: 3.39 ± 1.58 | Sex: F (1,47) 0.05, p = 0.8240  Stress: F (1,47) 3.88, p = 0.0547  Sex: Stress: F (1,47) 0.03, p = 0.8620 |
| Fig. 7F | Non-parametric | Female NS: 26.88 ± 11.21  Female stress: 19.83 ± 5.53  Male NS: 23.14 ± 13.73  Male stress: 24.54 ± 7.67 | Sex: F (1,47) 0.43, p = 0.518  Stress: F (1,47) 0.36, p = 0.552  Sex: Stress: F (1,47) 2.07, p = 0.157 |
| Fig. 7G | Non-parametric | Female NS: 9.13 ± 6.05  Female stress: 6.38 ± 2.82  Male NS: 9.19 ± 3.52  Male stress: 8.1 ± 3.95 | Sex: F (1,47) 1.39, p = 0.244  Stress: F (1,47) 2.63, p = 0.112  Sex: Stress: F (1,47) 0.00, p = 0.959 |
| Supplemental Fig. 1A | Non-parametric | Female NS: 2.44 ± 0.68  Female stress: 2.55 ± 0.82  Male NS: 2.81 ± 0.77  Male stress: 2.88 ± 1 | Sex: F (1,64) 1.78, p = 0.187  Stress: F (1,64) 0.00, p = 0.980  Sex: Stress: F (1,64) 0.00, p = 0.979 |
| Supplemental Fig. 1B | Non-parametric | Female NS: 1.84 ± 0.48  Female stress: 2.23 ± 1  Male NS: 2.16 ± 0.89  Male stress: 2.31 ± 1.62 | Sex: F (1,47) 0.20, p = 0.656  Stress: F (1,47) 0.92, p = 0.343  Sex: Stress: F (1,47) 0.35, p = 0.559 |
| Fisher’s exact test | | | |
| Figure |  |  | **P value** |
| Supplemental Fig. 2C |  |  | p = 0.7402 |
| Supplemental Fig. 2D |  |  | p = 0.3207 |
| T-test | | | |
| Figure | **Data structure** | **Mean (SD)** | **T-test** |
| Supplemental Figure 3A | Non-parametric | No PTX: 78.25 ± 91.47  PTX: 139.4 ± 182.4 | Mann-Whitney U = 244  N_1_ = 22  N_2_ = 25  p = 0.5191 |
| Supplemental Figure 3B | Non-parametric | No PTX: 8110 ± 11877  PTX: 13054 ± 18453 | Mann-Whitney U = 249  N_1_ = 22  N_2_ = 25  p = 0.5900 |
| Supplemental Figure 3C | Non-parametric | No PTX: 58.33 ± 47.89  PTX: 115.9 ± 151.8 | Mann-Whitney U = 229  N_1_ = 22  N_2_ = 25  p = 0.3353 |
| Supplemental Figure 3D | Non-parametric | No PTX: 77161 ± 79126  PTX: 111787 ± 111104 | Mann-Whitney U = 204  N_1_ = 20  N_2_ = 25  p = 0.3019 |
| Supplemental Figure 3E | Non-parametric | No PTX: 82.52 ± 109.7  PTX: 63.27 ± 113.9 | Mann-Whitney U = 89  N_1_ = 15  N_2_ = 19  p = 0.0655 |
| Supplemental Figure 3F | Non-parametric | No PTX: 7242 ± 10291  PTX: 6210 ± 13096 | Mann-Whitney U = 74  N_1_ = 15  N_2_ = 19  p = 0.0169 |
| Supplemental Figure 3G | Non-parametric | No PTX: 52.24 ± 37.45  PTX: 37.34 ± 44.54 | Mann-Whitney U = 94  N_1_ = 15  N_2_ = 19  p = 0.0963 |
| Supplemental Figure 3H | Non-parametric | No PTX: 64227 ± 71687  PTX: 37989 ± 69109 | Mann-Whitney U = 85  N_1_ = 15  N_2_ = 19  p = 0.0471 |
| Supplemental Figure 3I | Non-parametric | No PTX: 80.79 ± 183.1  PTX: 68.10 ± 35.20 | Mann-Whitney U = 121  N_1_ = 22  N_2_ = 25  p = 0.0007 |
| Supplemental Figure 3J | Parametric | No PTX: 224.8 ± 95.27  PTX: 160.6 ± 53.87 | t (32.24) = 2.791, p = 0.0088 |
| Supplemental Figure 3K | Non-parametric | No PTX: 62.09 ± 72.10  PTX: 64.36 ± 33.98 | Mann-Whitney U = 106  N_1_ = 15  N_2_ = 19  p = 0.2149 |
| Supplemental Figure 3L | Non-parametric | No PTX: 262.8 ± 144.0  PTX: 225.9 ± 115.9 | Mann-Whitney U = 130  N_1_ = 15  N_2_ = 19  p = 0.6812 |
