# Supplementary figures and images for: Sexual dimorphism and acute stress modulation of infralimbic-posterior hypothalamic synaptic transmission
Source: Front Cell Neurosci. 2026 Apr 10;20:1659293. doi: 10.3389/fncel.2026.1659293 (PMC13105900; doi:10.3389/fncel.2026.1659293)

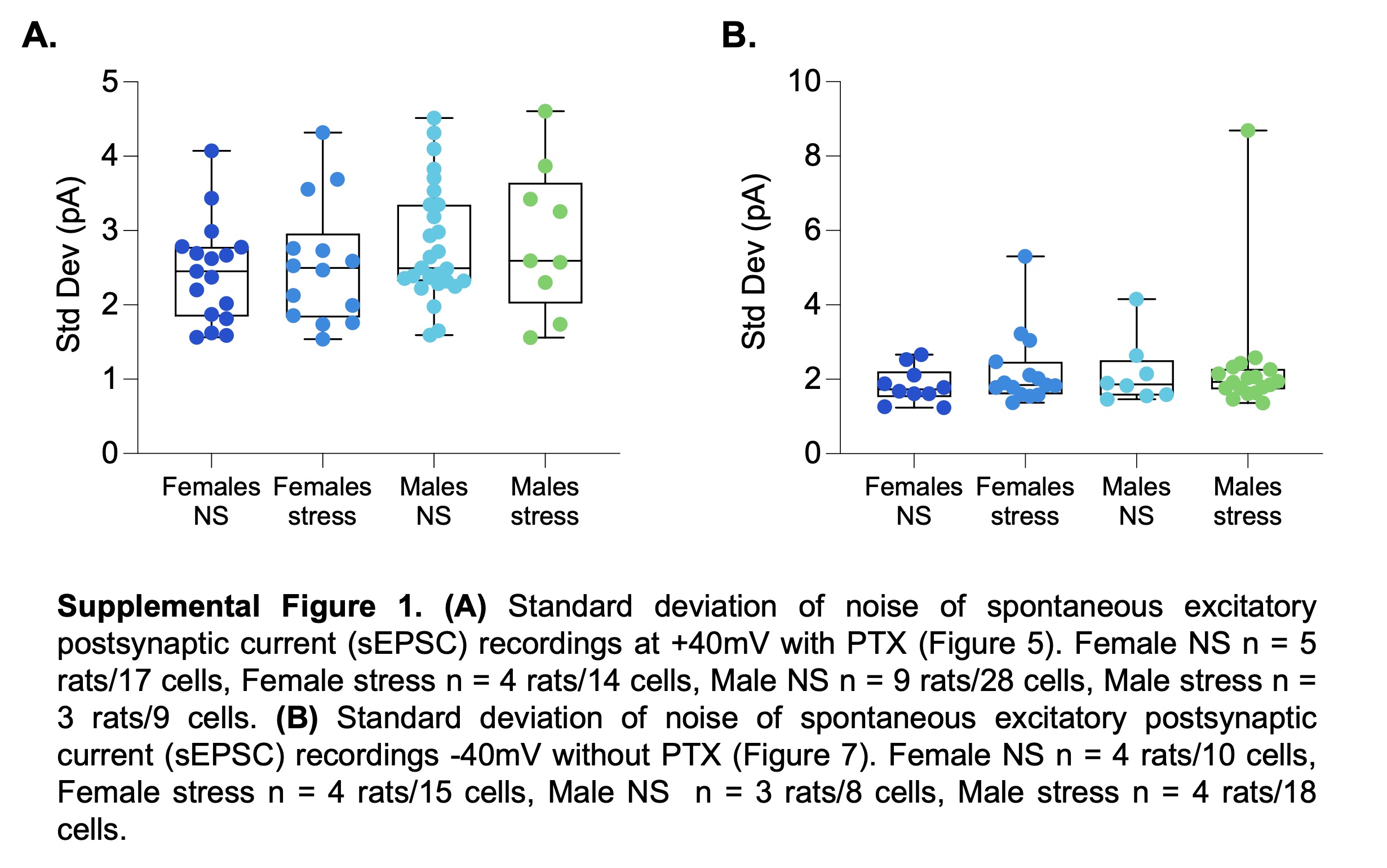

Supplement: Supplementary file 2 [file Image_1.jpg]

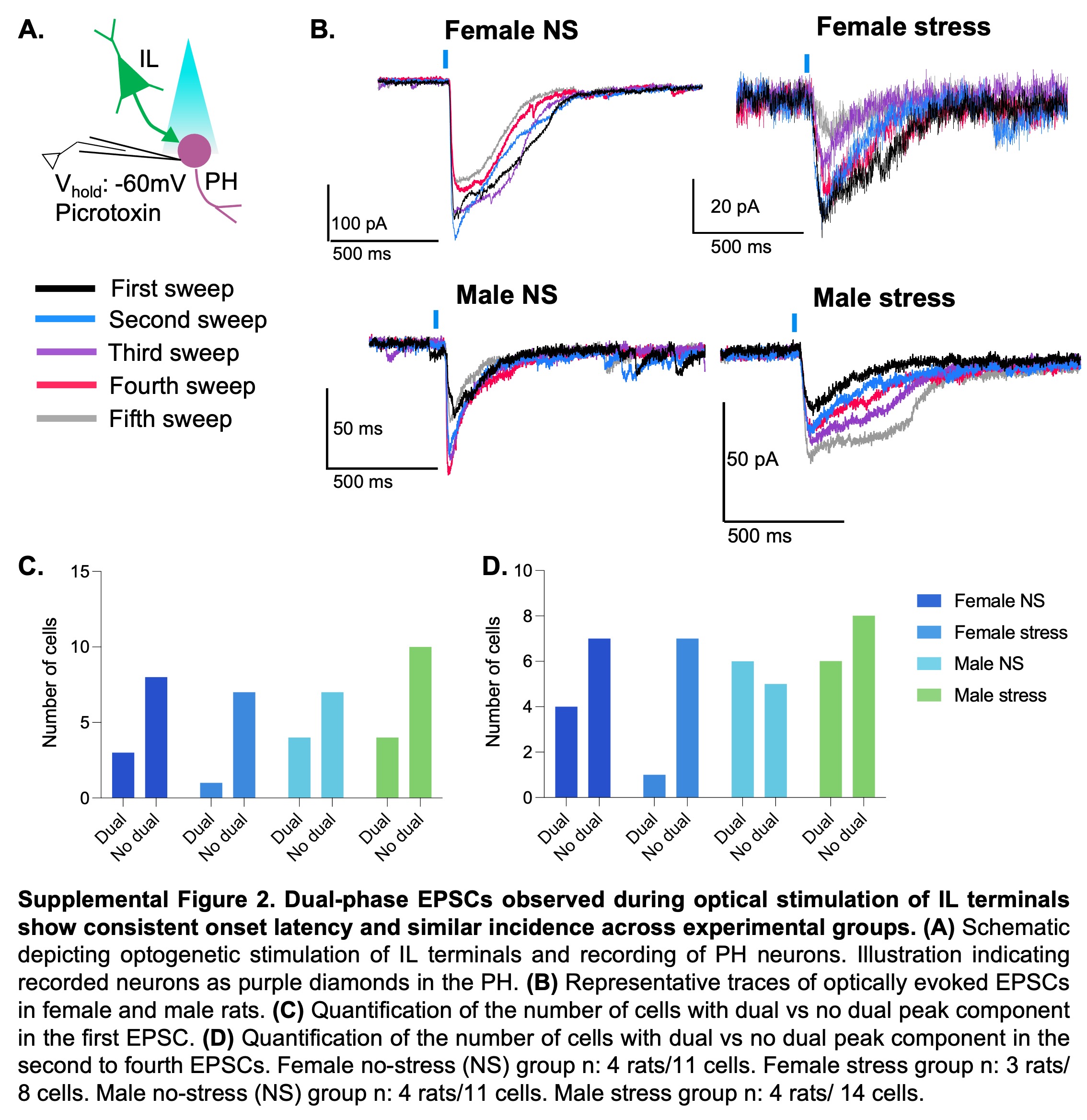

Supplement: Supplementary file 3 [file Image_2.jpg]

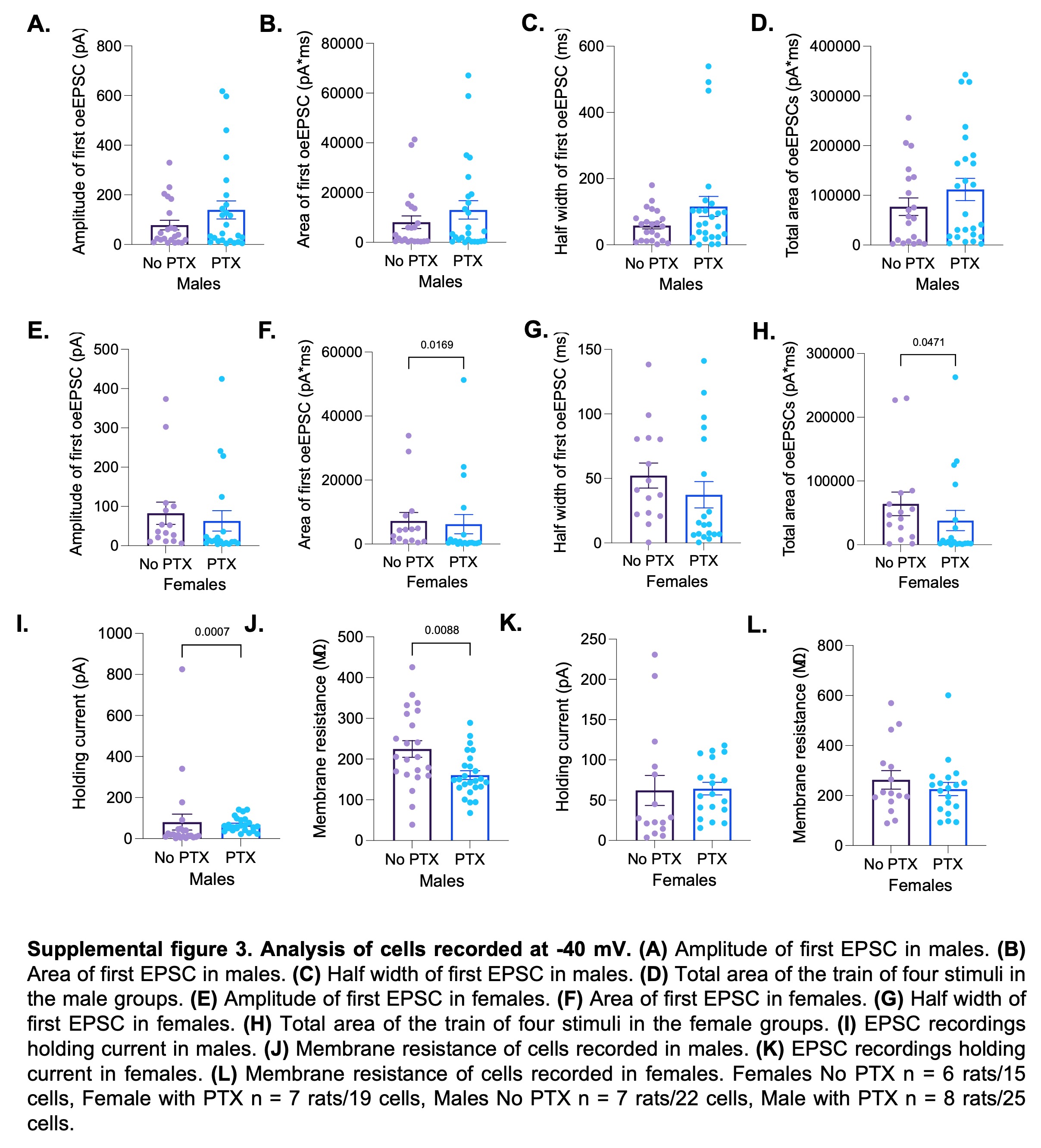

Supplement: Supplementary file 4 [file Image_3.jpeg]
